# Interface‐Enhanced Electrochemical Epoxidation Over a Core‐Shell Nanozeolite TS‐1@Mn‐N‐C Catalyst

**DOI:** 10.1002/advs.77922

**Published:** 2026-09-27

**Authors:** Wenbiao Zhang, Guanqiao Zhang, Wanling Zhang, Kun Yu, Di Pan, Zhuxin Gui, Yi Tang, Qingsheng Gao

**Affiliations:** ^1^ College of Chemistry and Materials Science State Key Laboratory of Bioactive Molecules and Druggability Assessment and Guangdong Basic Research Center of Excellence for Natural Bioactive Molecules and Discovery of Innovative Drugs Jinan University Guangzhou China; ^2^ Department of Chemistry College of Smart Materials and Future Energy State Key Laboratory of Porous Materials for Separation and Conversion Shanghai Key Laboratory of Molecular Catalysis and Innovative Materials and Laboratory of Advanced Materials Fudan University Shanghai China

**Keywords:** electrochemical epoxidation, interfaces, Mn‐N‐C, tandem reactions, Ti‐Si zeolites

## Abstract

Conventional olefin epoxidation suffers from poor atomic economy and significant environmental/economic burdens due to the reliance on harsh oxidants. While electrochemical epoxidation using water as an oxygen source presents a sustainable alternative, existing systems remain hampered by noble‐metal dependence or inefficiency. To address this, we design a core‐shell nanostructure integrating titanium silicalite‐1 (TS‐1) nanozeolite with Mn‐N‐C (TS‐1@Mn‐N‐C). Critically, the interfacial synergy drives a tandem catalytic process: Mn‐N‐C sites electrosynthesize H_2_O_2_ via 2e^−^ water oxidation, which directly migrates to adjacent TS‐1 active centers for selective epoxidation. Such spatial coupling ensures maximized H_2_O_2_ utilization and enhanced catalytic efficiency. As expected, TS‐1@Mn‐N‐C achieves exceptional performance for the epoxidation of cyclooctene to 1,2‐epoxycyclooctane in neutral electrolytes, outperforming both individual components and recently reported benchmarks. In situ characterizations combined with theoretical calculations reveal that the TS‐1@Mn‐N‐C interface lowers the energy barrier for H_2_O_2_ activation toward reactive η‐Ti‐OOH intermediates, enabling efficient and sustained epoxidation. The catalyst also shows broad substrate applicability, highlighting its potential as a sustainable and cost‐effective solution for electrochemical olefin epoxidation. This work pioneers a dual‐site interface strategy to optimize reaction kinetics and advance green chemical synthesis.

## Graphical abstract

Core–shell interface of TS‐1@Mn‐N‐C enables in situ generation of high‐concentration H_2_O_2_ and its spatially confined activation toward epoxidation‐active *η*‐Ti‐OOH species, leading to highly efficient and continuous electrochemical epoxidation. The work successfully addresses the key challenge of inefficient mass transfer and slow kinetics caused by spatial separation in electrochemical‐chemical tandem catalysis.

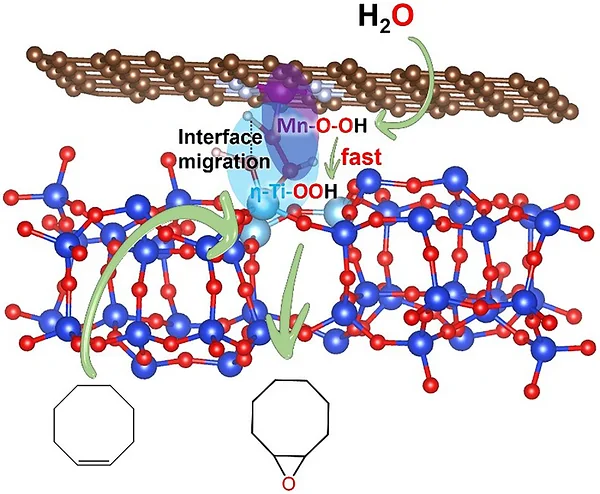

## 1 Introduction

Olefin epoxidation is a fundamental process in chemical industries, as the resulting epoxides serve as essential intermediates for the production of epoxy resins, coatings, surfactants, pharmaceuticals, food additives, fragrances, etc. [1, 2, 3]. Currently, the industrial synthesis of epoxides primarily relies on the chlorohydrin process and catalytic aerobic or peroxide‐based oxidation [4, 5, 6], which suffer from several critical drawbacks, including harsh reaction conditions, high energy consumption, complex operational procedures, and significant safety risks. Meanwhile, they contribute substantially to carbon emissions due to the reliance on fossil fuel‐derived reagents and energy sources [7, 8]. There is an urgent need to develop cost‐effective and eco‐friendly alternatives that adhere to green chemistry principles and support carbon neutrality [9, 10].

Electrocatalysis powered by sustainable electricity offers a compelling alternative to the traditional olefin epoxidation approaches, featuring mild reaction conditions, high energy efficiency, and the use of water as a green oxygen source [11, 12]. Direct electrooxidation of olefins typically employs noble‐metal catalysts such as Pt, Pd, Ag, and their oxides [13, 14, 15, 16], which often yield not only epoxides but also aldehydes or carboxylic acids as byproducts [17, 18]. To circumvent the use of precious metals, manganese oxides have been explored as cost‐efficient alternatives; however, their application is severely limited by the low Faradaic efficiency (F.E. < 40%) for epoxidation [19], even after doping with a precious metal like Ir [20]. Strategies combining mediated reactions can significantly enhance epoxidation by generating reactive intermediates, such as HOX (X = Cl and Br) from halides, which react with olefins to produce target products and subsequently revert to their original states [21, 22, 23, 24]. Compared to environmentally hazardous halide mediators, the in situ generation of peroxides would enable more benign oxidation under the assistance of epoxidation‐efficient components like titanium silicalite‐1 (TS‐1). TS‐1, with a MFI zeolite topology, is highly effective for H_2_O_2_‐mediated epoxidation, producing high‐purity epoxides with minimal side reactions [25]. Recent studies have highlighted its application in electrochemical epoxidation, such as coupling the 2‐electron oxygen reduction reaction (2e^−^ ORR) at the cathode with dispersed TS‐1 in electrolytes [26, 27, 28]. However, these systems struggle to fully utilize high‐concentration intermediates at electrode/electrolyte interfaces due to the spatial separation of H_2_O_2_ synthesis and organic oxidation, unfortunately leading to greater limitation from sluggish mass transfer and inevitable H_2_O_2_ dilution [29]. Moreover, the low solubility of gaseous O_2_ in electrolytes significantly restricts H_2_O_2_ generation at gas‐liquid‐solid triple‐phase interfaces [30], adversely affecting the overall reaction performance. In contrast, H_2_O_2_ production via the 2‐electron water oxidation reaction (2e^−^ WOR) could offer a more efficient pathway for coupling with TS‐1 [31], which, however, remains unexplored. Designing dual‐site interfaces that synergistically integrate H_2_O_2_ electrosynthesis via 2e^−^ WOR and subsequent H_2_O_2_ activation for epoxidation, while maximizing interfacial concentrations, is critical for advancing electrochemical olefin epoxidation.

Herein, we designed a core‐shell nanostructure composed of TS‐1 zeolite encapsulated by Mn‐N‐C (TS‐1@Mn‐N‐C), which orchestrated a tandem process at the interfaces: the H_2_O_2_ generated on Mn‐N‐C sites via 2e^−^ WOR undergoes spatially confined transfer to TS‐1 centers for the selective epoxidation of olefins. TS‐1@Mn‐N‐C demonstrated exceptional performance for cyclooctene epoxidation in 0.1 m LiClO_4_ (pH 6.8), where pH‐neutral conditions effectively suppressed undesired side‐reactions (e.g., epoxide hydration and oxygen evolution) [32, 33]. At 2.0 V vs. Ag/AgCl, it achieved superior 1,2‐epoxycyclooctane selectivity (83.0%) and yield (64.7%), significantly outperforming both TS‐1 alone (70.8% selectivity, 17.7% yield) and Mn‐N‐C (64.2% selectivity, 45.0% yield). Combined experimental and theoretical investigations confirmed the synergistic interplay between H_2_O_2_ production and cyclooctene epoxidation at the TS‐1@Mn‐N‐C interfaces. Consequently, the Mn‐N‐C sites facilitated 2e^−^ WOR to H_2_O_2_, while the TS‐1@Mn‐N‐C interface significantly reduced the energy barrier for H_2_O_2_ activation and promoted the formation of reactive η‐Ti‐OOH intermediates essential for the epoxidation. This synergy minimizes the need for long‐range H_2_O_2_ diffusion and bulk accumulation by promoting its rapid utilization at the adjacent Ti site. Moreover, TS‐1@Mn‐N‐C exhibited broad applicability for the epoxidation of cyclic and chain olefins, underscoring its potential for practical electrosynthesis.

## 2 Results and Discussion

### 2.1 Materials Synthesis and Characterization

**FIGURE 1:**
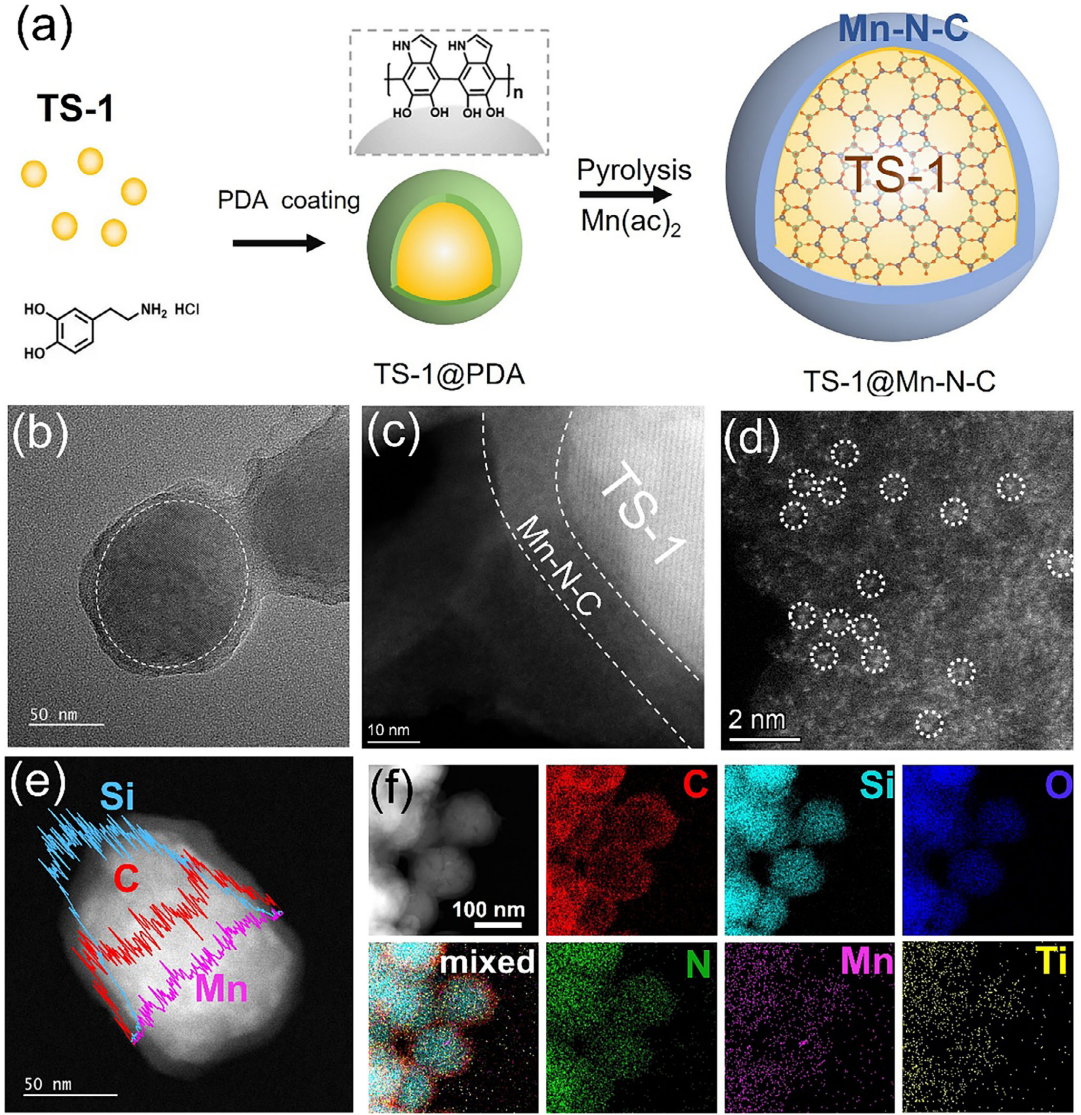
(a) Schematic illustration for the fabrication of TS‐1@Mn‐N‐C nanocomposites. (b) TEM, (c) HR‐TEM, (d) HAADF‐STEM, (e) linear‐EDS images, and (f) EDS‐mapping of TS‐1@Mn‐N‐C.

Initial screening of M‐N‐C electrocatalysts (M = Pd, Ag, Mn, Zn, Ni, and Co) for H_2_O_2_ electrosynthesis revealed Mn‐N‐C as the top performer (Figure S1). Afterward, the composite of TS‐1@Mn‐N‐C was fabricated via pyrolyzing polydopamine (PDA) coated TS‐1 (TS‐1@PDA) with a manganese acetylacetonate (Mn(acac)_2_) precursor under an inert atmosphere (Figure 1a). PDA coating was achieved on nanosized TS‐1 zeolites (∼ 100 nm) via the polymerization of dopamine monomers in a Tris‐HCl buffer, resulting in core‐shell TS‐1@PDA that was then dispersed into Mn(acac)_2_ solution. After the pyrolysis at 800°C, TS‐1@Mn‐N‐C nanocomposites were obtained. Fourier transform infrared (FT‐IR) spectroscopy and thermogravimetric analysis (TGA) were conducted to explore the above coating and pyrolysis process. In FT‐IR (Figure S2), the band at 1620 cm^−1^ on TS‐1@PDA was attributed to the vibration of a benzene ring, indicative of the encapsulation by PDA. Accordingly, the TGA showed the gradual weight loss of TS‐1@PDA from 100 to 800°C (Figure S3), associated with the carbonization of the external PDA layer [34].

Scanning electron microscopy (SEM) showed negligible morphological differences among TS‐1, TS‐1@PDA, and TS‐1@Mn–N–C (Figure S4). As further identified by transmission electron microscopy (TEM, Figure 1b), there was a clear boundary (the white dashed line) between the TS‐1 core and the Mn–N–C coating. The *d*‐spacing of 1.11 nm observed on the core corresponded to the (200) lattice of TS‐1, while no lattice fringes could be discriminated in the Mn–N–C layer (Figure 1c). The aberration‐corrected high‐angle annular dark field scanning transmission electron microscopy (AC‐HAADF‐STEM) revealed predominantly atomically dispersed Mn with bright spots in both TS‐1@Mn‐N‐C and Mn‐N‐C samples (Figure 1d and Figure S5), indicating that isolated Mn sites constitute the major population of Mn species. This atomic‐level dispersion of Mn is critical for enhancing catalytic activity and stability, as it allows for more active sites exposed for reactions. The energy dispersive x‐ray spectroscopy (EDS) linescan (Figure 1e) and the corresponding mapping (Figure 1f) confirmed a core‐shell structure, with distinct elemental distribution patterns highlighting the core (Si, Ti, and O) and shell (C, N, and Mn) regions.

Furthermore, x‐ray diffraction (XRD) analysis presented the well‐preserved MFI topology of TS‐1 after pyrolysis (Figure S6), with the characteristic diffractions at 2*θ* = 7.8°, 8.8°, 23.2°, 23.8°, and 24.3° [35, 36]. X‐ray photoelectron spectroscopy (XPS) was then performed to analyze the chemical states of elements. The Mn 2p spectra confirmed the co‐presence of Mn^2+^, Mn^3+^, and Mn^4+^ in both TS‐1@Mn‐N‐C and Mn‐N‐C (Figure S7) [37]. Meanwhile, the N 1s spectra revealed characteristic pyridinic‐N, pyrrolic‐N, and Mn‐N species (Figure S8), while the O 1s profile showed Mn(VI)‐O features attributed to surface oxidation (Figure S9) [38, 39]. Compared to zeolite materials with poor conductivity, TS‐1@Mn‐N‐C exhibited greatly improved conductivity after carbon coating (Figure S10), conducive to the following electrochemical epoxidation. Argon adsorption‐desorption isothermals were carried out to characterize the pore structure of the different samples (Figure S11). TS‐1@Mn–N–C exhibited a typical type I‐B isotherm with a high surface area and large pore volume, suggesting that the porous Mn–N–C layers would not hinder substrate molecules from reaching the internal active sites of the TS‐1 core. Moreover, the pore distribution of TS‐1 remained unchanged in TS‐1@Mn‐N‐C associated with the well‐maintained framework.

**FIGURE 2:**
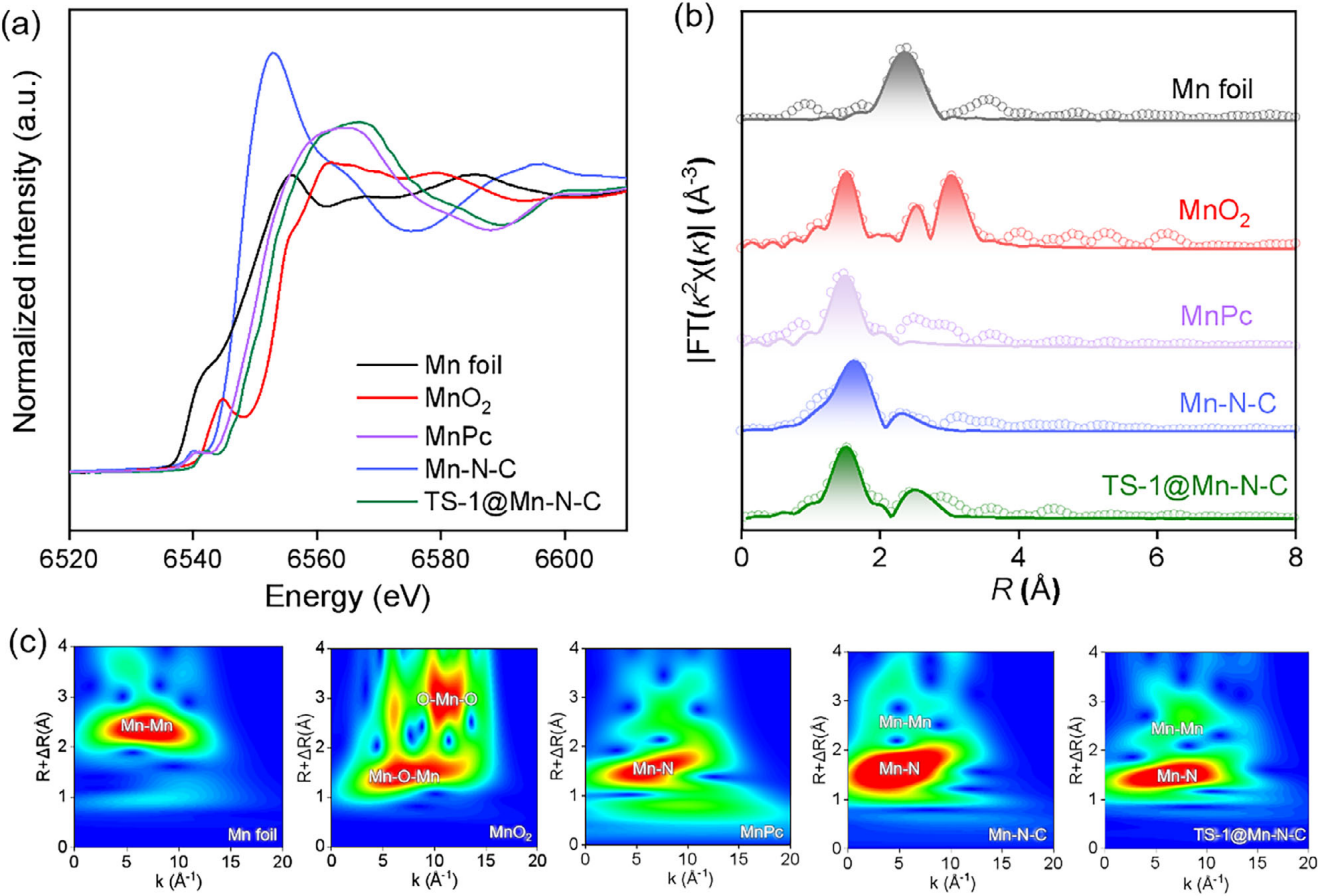
(a) Mn K‐edge XANES, (b) FT‐EXAFS, and (c) the corresponding WT of TS‐1@Mn‐N‐C, Mn‐N‐C, and Mn foil, MnO_2_ and MnPc references.

To further understand the coordination environment and valence state of Mn in TS‐1@Mn‐N‐C and Mn‐N‐C, x‐ray absorption near‐edge structure (XANES) and extended x‐ray absorption fine structure (EXAFS) were analyzed, along with three references of Mn foil, MnO_2,_ and Mn phthalocyanine (MnPc). The Mn K‐edge XANES results (Figure 2a and Table S1) showed that the absorption edge energies of Mn‐N‐C and TS‐1@Mn‐N‐C samples fell between those of Mn foil and MnO_2_, suggesting an average oxidation state between 0 and +4. Compared to Mn‐N‐C, TS‐1@Mn‐N‐C exhibited a distinct blue shift in the Mn K‐edge absorption, with its position closer to the MnPc reference. The results indicated that the spatial confinement by TS‐1 promotes a more ordered Mn‐N coordination environment, consistent with the Mn‐N coordination observed in MnPc, whereas free Mn‐N‐C likely adopts non‐planar disordered configurations. Notably, the slightly higher absorption edge energy of TS‐1@Mn‐N‐C compared to MnPc suggested strong interfacial interactions with the TS‐1 framework. To further clarify the local coordination environment of Mn, the Mn K‐edge EXAFS spectra were re‐fitted using a Mn‐N first‐shell contribution together with an additional Mn‐related second‐shell scattering path. To illustrate the local coordination environment, the prominent Fourier‐transformed k3‐weighted EXAFS (FT‐EXAFS) was primarily assigned to the Mn‐N first coordination shell (Figure 2b and Figure S12). TS‐1@Mn‐N‐C and Mn‐N‐C presented a prominent peak at 1.55 and 1.60 Å, respectively, corresponding to Mn‐N bonds, and a weaker second‐shell Mn‐related contribution (∼2.30 Å). These results indicate that Mn‐N coordination constitutes the dominant local environment, while a minor fraction of Mn species may experience secondary Mn‐related interactions. Although the weak Mn‐related second‐shell contribution suggests the presence of minor Mn–Mn interactions, this feature is shared by the compared Mn‐containing catalysts. The Mn‐N scattering path in TS‐1@Mn‐N‐C closely matched that of the MnPc reference, consistent with the XANES results. Therefore, the Mn sites in TS‐1@Mn‐N‐C are more appropriately described as predominantly atomically dispersed rather than exclusively isolated. Such secondary Mn‐related interactions may further modulate the electronic structure of the Mn sites and thus influence H_2_O activation and H_2_O_2_ generation. Accordingly, we performed a wavelet transform (WT) analysis to achieve higher resolution in R‐space (Figure 2c). The Mn‐N (1.55 Å) scattering path of TS‐1@Mn‐N‐C observed in R space was shorter than that of Mn‐N‐C (1.60 Å), indicating again the strong interfacial interactions. This is conducive to the tandem electrochemical‐chemical processes on the adjacent Mn and Ti sites.

### 2.2 Electrochemical Epoxidation Performance and Mechanism

**FIGURE 3:**
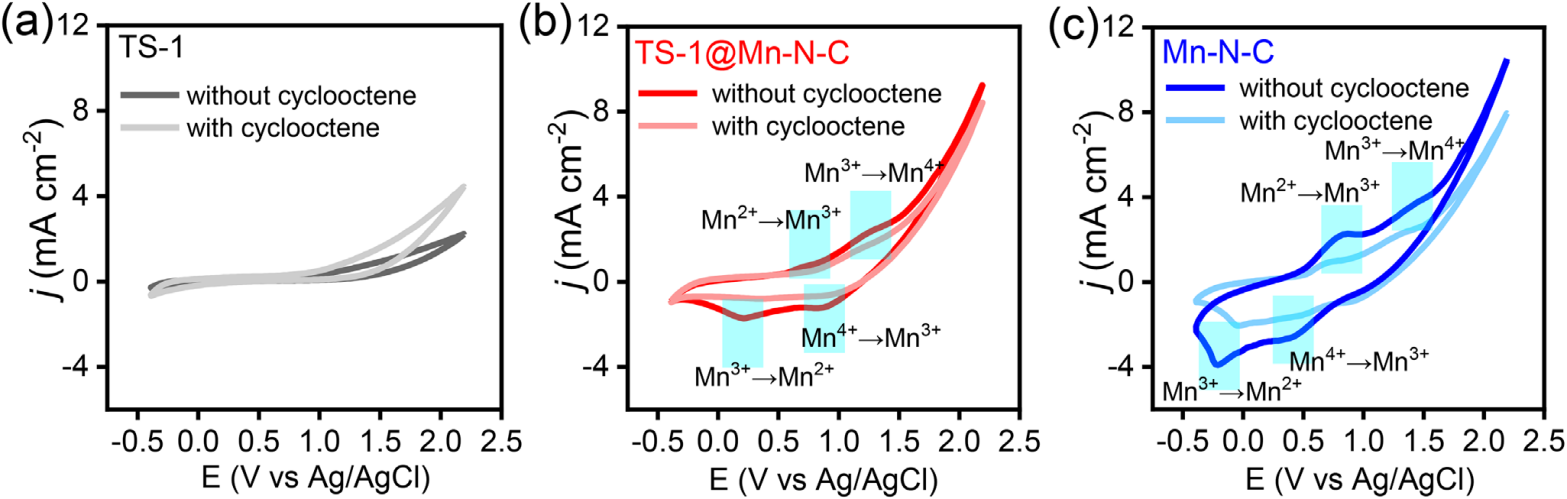
CV curves of (a) TS‐1, (b) TS‐1@Mn‐N‐C, and (c) Mn‐N‐C in 0.1 m LiClO_4_ (pH 6.8) with/without 10 mm cyclooctene.

Before conducting electrochemical epoxidation, we characterized the cyclic voltammetry (CV) profiles of TS‐1, TS‐1@Mn‐N‐C, and Mn‐N‐C in 0.1 m LiClO_4_ (pH 6.8). The TS‐1 modified electrode exhibited a small current regardless of the presence or absence of cyclooctene (Figure 3a), indicating negligible electrocatalytic activity. By contrast, TS‐1@Mn‐N‐C and Mn‐N‐C showed obvious redox peaks in the CVs (Figure 3b,c). In the absence of cyclooctene, TS‐1@Mn‐N‐C displayed the reduction of Mn^4+^→Mn^3+^ and Mn^3+^→Mn^2+^ at more positive potentials than Mn‐N‐C [40, 41], indicating the higher activity of Mn sites strongly interacted with TS‐1. Upon the addition of cyclooctene, the redox peaks were obviously weakened, as cyclooctene epoxidation on the catalyst surface consumed the reactive oxygen species (ROS) that were originally responsible for the Mn‐related redox process.

**FIGURE 4:**
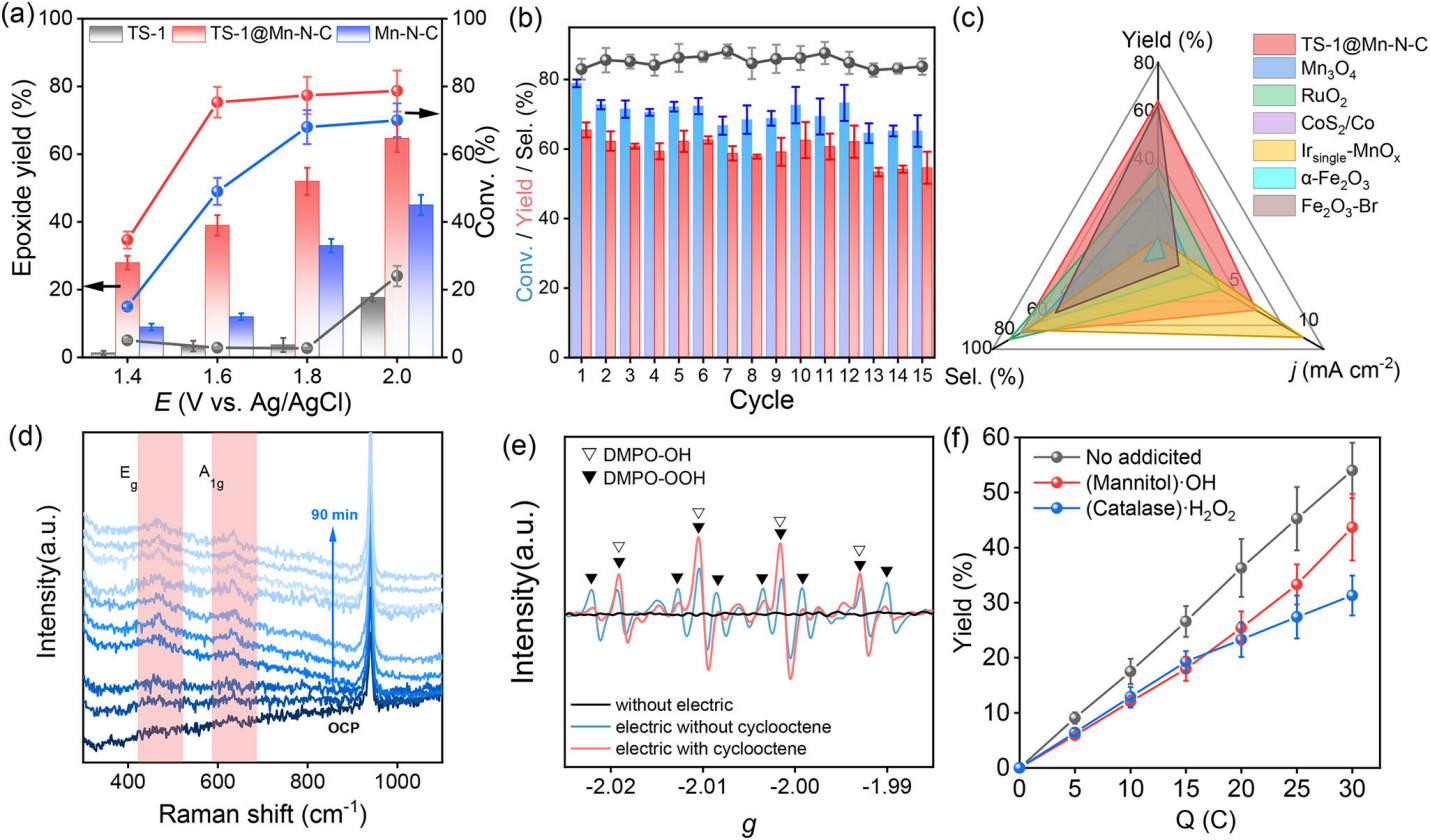
(a) Epoxidation performance over TS‐1@Mn‐N‐C, Mn‐N‐C, and TS‐1 at 1.4 ∼ 2.0 V vs. Ag/AgCl in 0.1 m LiClO_4_ with 10 mm cyclooctene, and (b) cycling stability of TS‐1@Mn‐N‐C over 15 consecutive cycles at 2.0 V vs. Ag/AgCl. (c) Comparison between TS‐1@Mn‐N‐C with recent reports (Table S2) for the electrochemical epoxidation of cyclooctene. (d) In situ Raman spectra of TS‐1@Mn‐N‐C collected during cyclooctene epoxidation at 2.0 V vs. Ag/AgCl and (e) in situ EPR spectra of the analyte collected with DMPO as the trapping agent at 2.0 V vs. Ag/AgCl. (f) Charge‐yield curves of epoxidation yield on TS‐1@Mn‐N‐C with different quenchers.

Afterward, the epoxidation performance was evaluated at 1.4 ∼ 2.0 V vs. Ag/AgCl in a 0.1 m LiClO_4_ aqueous mixed solution with 10 mm cyclooctene (Figure 4a). The conversion and epoxide yield followed the order of TS‐1@Mn‐N‐C > Mn‐N‐C > TS‐1. In contrast to bare TS‐1 with negligible activity, TS‐1@Mn‐N‐C and Mn‐N‐C presented obvious cyclooctene conversion and 1,2‐epoxycyclooctane yield, suggesting the key contribution to electrochemical epoxidation by Mn‐N‐C sites. Although TS‐1@Mn‐N‐C only exhibited a slightly higher cyclooctene conversion than Mn‐N‐C, the yield of the target 1,2‐epoxycyclooctane was 2–3 fold higher at low potentials (1.4 and 1.6 V) and 30%–40% higher at high potentials (1.8 and 2.0 V), associated with the selective epoxidation enabled by the core‐shell interfaces. At 2.0 V vs. Ag/AgCl, it achieved superior selectivity of 83.0% and yield of 64.7%, significantly outperforming both TS‐1 alone (70.8% selectivity, 17.7% yield) and Mn‐N‐C (64.2% selectivity, 45.0% yield). Notably, under these conditions, the TS‐1@Mn‐N‐C and Mn‐N‐C electrodes show comparable impedance behavior (Figures S13 and S14), which indicates that the higher epoxide yield of TS‐1@Mn‐N‐C cannot be attributed solely to enhanced conductivity. The time‐dependent conversion of cyclooctene to 1,2‐epoxycyclooctane on TS‐1@Mn‐N‐C was in accordance with the visible decrease of cyclooctene (Figure S15), while the by‐product cyclooctanone, derived from the electrooxidation, was limited to a low level. Moreover, TS‐1@Mn‐N‐C afforded good recyclability for epoxidation with well‐retained conversion (> 70%), product selectivity (> 80%), and yield (> 55%) over 15 consecutive cycles (Figure 4b and Figure S16). The structural stability of TS‐1@Mn‐N‐C was further corroborated by the post‐reaction characterizations (XRD, TEM, and x‐ray absorption spectroscopy), which clearly identified the well‐preserved core‐shell structure with the consistent electronic/coordination environment (Figures S17–S19). In further comparison with recently reported catalysts for the (photo)electrochemical epoxidation of cyclic olefins [19, 20, 21, 42, 43, 44], TS‐1@Mn‐N‐C presented an outstanding overall performance with excellent selectivity and yield (Figure 4c and Table S2).

In situ Raman spectroscopy monitoring of the epoxidation reaction revealed characteristic Mn‐O bending (E_g_) and stretching (A_1g_) vibrations at ∼474.4 and ∼645.1 cm^−1^ (Figure 4d) [45], respectively. The progressive intensification of these bands with reaction time indicated continuous accumulation of Mn‐O species. Accordingly, EPR trapping experiments with 5,5‐dimethyl‐1‐pyrroline N‐oxide (DMPO) showed the presence of •OH and •OOH radicals (Figure 4e), which were derived from the initial step of 2e^−^ WOR and the H_2_O_2_ activation at Ti sites, respectively. Upon adding cyclooctene, the •OOH signal decreased sharply, indicating consumption by Ti‐OOH mediated epoxidation. Concurrently, •OH signals slightly intensified, suggesting accelerated H_2_O_2_ generation and thus •OH formation due to the •OOH depletion. Quenching experiments with scavengers further supported this assumption (Figure 4f). The addition of catalase, which decomposes H_2_O_2_ caused a pronounced activity drop, as it directly reduced the key epoxidizing species of Ti‐OOH. In contrast, mannitol, as a •OH scavenger, had a weaker inhibitory effect, suggesting the activation of H_2_O_2_ as the rate‐determining step of epoxidation [46].

Accordingly, the time‐dependent measurements of H_2_O_2_ concentration using Mn‐N‐C and TS‐1@Mn‐N‐C, both in the absence and presence of cyclooctene, support a tandem process in which H_2_O_2_ is electrochemically generated at Mn‐N‐C and subsequently consumed at the TS‐1 component for cyclooctene epoxidation (Figure S20). The pronounced suppression of the steady‐state H_2_O_2_ concentration in the presence of cyclooctene indicates that electrogenerated H_2_O_2_ is rapidly consumed in the subsequent epoxidation reaction. Notably, this effect is much more significant for TS‐1@Mn‐N‐C than for Mn‐N‐C, supporting the role of TS‐1 in promoting efficient H_2_O_2_ utilization. Meanwhile, the low epoxidation activity of pristine M‐N‐C catalysts (M = Pd, Mn, Zn, Ni, Co, and Ag) further identified the active sites of TS‐1 for cyclooctene epoxidation (Figure S21). We next examined the influence of electrolyte composition (i.e., 0.1 m LiClO_4_, 0.05 m Na_2_SO_4_, and 0.1 m NaHCO_3_) to determine whether the conditions favoring H_2_O_2_ production are also optimal for epoxide formation (Figure S22). Although the H_2_O_2_ production rate increased in the order LiClO_4_ < Na_2_SO_4_ < NaHCO_3_, the corresponding epoxide yields did not follow the same trend. Notably, LiClO_4_ afforded the highest epoxide yield, despite exhibiting the lowest H_2_O_2_ production rate among the three electrolytes. These results indicate that bulk H_2_O_2_ generation alone does not determine the overall epoxidation efficiency. Instead, rapid interfacial utilization of electrogenerated H_2_O_2_ by the adjacent TS‐1 sites plays a critical role in directing the reaction toward epoxidation. These results collectively demonstrate that H_2_O_2_ is the crucial ROS for the epoxidation, generated electrochemically via Mn‐N‐C and subsequently activated by TS‐1 to form Ti‐OOH, the active species for epoxidation [47].

**FIGURE 5:**
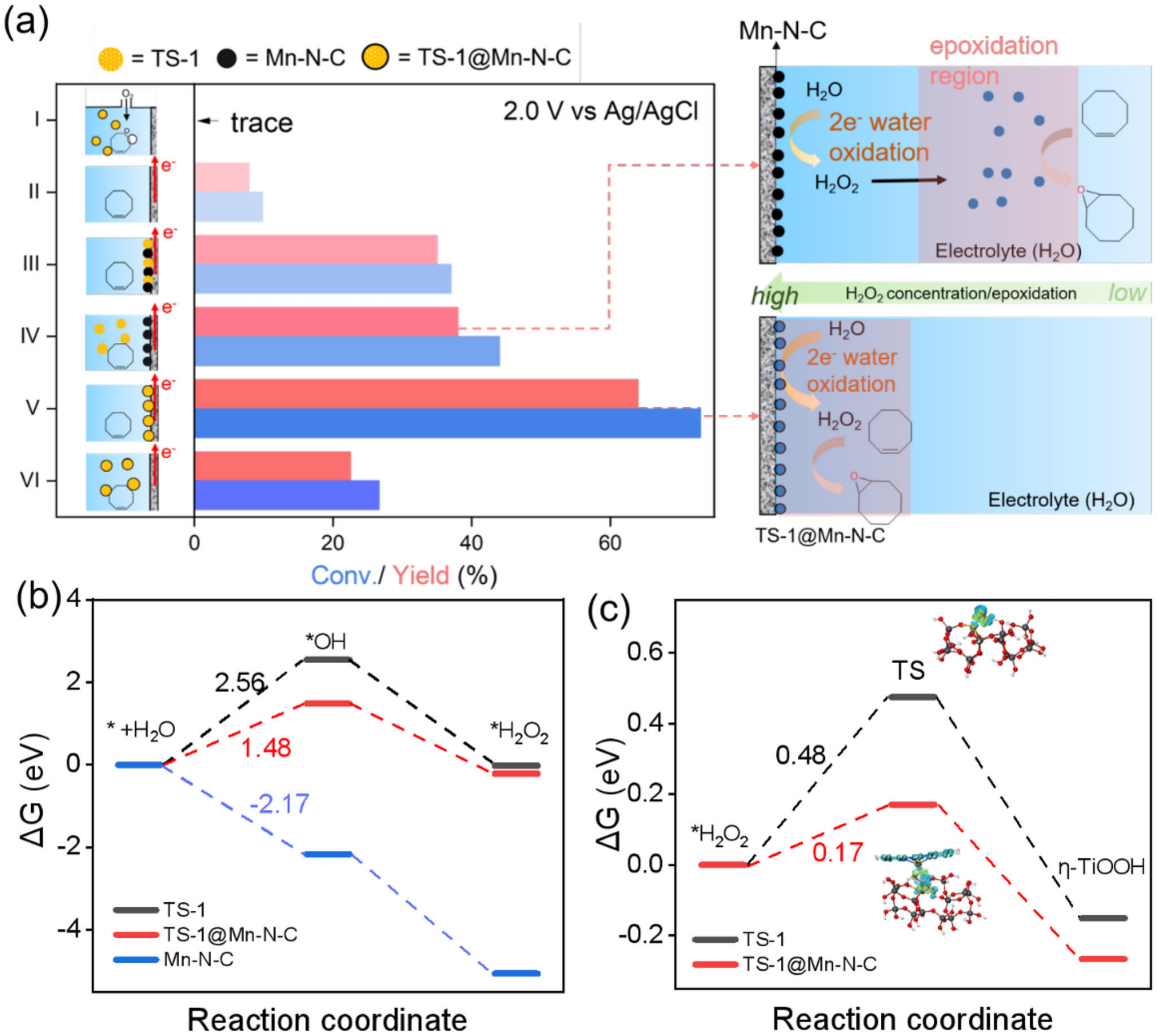
(a) Scheme of controlled reaction conditions (I: TS‐1@Mn‐N‐C in electrolyte, 0.1 MPa O_2_; II: carbon cloth, 2.0 V vs. Ag/AgCl; III: TS‐1 mixed with Mn‐N‐C physically; IV: TS‐1 in electrolyte, Mn‐N‐C on electrode, 2.0 V vs. Ag/AgCl; V: TS‐1@Mn‐N‐C on electrode, 2.0 V vs. Ag/AgCl; VI: TS‐1@Mn‐N‐C in electrolyte, 2.0 V vs. Ag/AgCl, the Ti and Mn content was controlled as the same in Cases III‐VI. DFT calculated Gibbs free energy diagram for (b) 2e^−^ WOR and (c) H_2_O_2_ activation.

To further investigate the coupled H_2_O_2_ electrosynthesis and epoxidation processes at TS‐1@Mn‐N‐C interfaces, we conducted a series of control experiments (Figure 5a). Neither TS‐1 powder alone in O_2_‐saturated electrolyte (without bias, Case I) nor blank carbon cloth at 2.0 V vs. Ag/AgCl (Case II) exhibited notable activity (conversion < 10%), confirming the negligible background interference. Importantly, a physically mixed TS‐1/Mn‐N‐C electrode (Case III) afforded only 39% conversion and 35% epoxide yield. When Mn‐N‐C was coated on the electrode while TS‐1 was dispersed in the electrolyte (Case IV), the electrogenerated H_2_O_2_ at anode diffused randomly through the bulk electrolyte to reach TS‐1, resulting in the markedly lower conversion (44.0%) and yield (38.1%) compared to TS‐1@Mn‐N‐C (77.0% and 64.7%) deposited on the electrode (Case V). Since this electrochemical process primarily occurs at the electrode surface, dispersing TS‐1@Mn‐N‐C in the anolyte inevitably reduced activity (Case VI). The above comparison highlights the critical synergy between Mn‐N‐C and TS‐1: Mn‐N‐C electrogenerates H_2_O_2_ and rapidly transfers it to adjacent TS‐1, where the Ti sites confine and activate H_2_O_2_ for epoxidation. TS‐1 is indispensable because direct olefin epoxidation without H_2_O_2_ activation faces high energy barriers and ill‐defined pathways [48]. Increasing the size of the TS‐1 core led to a slight decrease in the 1,2‐epoxycyclooctane yield (Figure S23), suggesting that the epoxidation occurs predominantly at the TS‐1/Mn‐N‐C interface. Replacing TS‐1 with zeolites lacking Ti sites, such as S‐1 or ZSM‐5, resulted in a significant decline in reaction activity (Figure S24), confirming again the indispensable Ti sites for epoxidation. Moreover, the efficiency of electrochemical epoxidation was governed by the kinetic matching between the electrocatalytic H_2_O_2_ generation on the Mn‐N‐C shell and the chemical epoxidation on the TS‐1 core, with notable dependence on the relative concentrations of water and cyclooctene [16, 19, 20]. As the water concentration increased, the rate of H_2_O_2_ production and the corresponding epoxidation current both increased (Figure S25a), likely attributable to the enhanced electrolyte conductivity. Meanwhile, increasing the substrate concentration led to a decrease in the epoxidation current, which can be explained by excessive adsorption of the substrate at the Mn‐N‐C shell, inhibiting the 2e^−^ WOR pathway (Figure S25b).

As a direct comparison, the thermal catalytic system using either TS‐1 or TS‐1@Mn‐N‐C required as high as 7.5 wt.% H_2_O_2_ concentration, a staggering 75‐fold higher than that produced in the electrocatalytic system with TS‐1@Mn‐N‐C, to achieve the same product yield under identical reaction conditions (Figure S26). This striking contrast vividly demonstrates the superior efficiency enabled by the interfacial synergism for the epoxidation. Notably, the electrocatalytic system with TS‐1@Mn‐N‐C offers additional advantages, including facile catalyst recovery and continuous operation capability through successive substrate additions to the electrolyte, enabled by the in situ H_2_O_2_ generation via 2e^−^ WOR. In cycling tests (Figure S27), the electrocatalytic system exhibited progressively enhanced activity, ultimately surpassing the performance of conventional TS‐1‐H_2_O_2_ thermo‐catalysis after five consecutive runs without electrolyte replacement. These findings confirm that the electrochemical epoxidation maintains stable efficiency despite the consumption of ROSs, attributable to the continuous electrosynthesis and efficient utilization of H_2_O_2_.

Density functional theory (DFT) calculations were further conducted to acquire deeper mechanistic insights into the tandem H_2_O_2_ electrosynthesis‐chemical epoxidation occurring at the TS‐1@Mn‐N‐C interfaces (Figure 5b,c). The calculations mapped the Gibbs free energy landscape for the reaction pathway leading to the formation of the key epoxidation species, *η*‐Ti‐OOH [25].

The computational model was constructed to represent the predominant Mn–N‐coordinated environment identified experimentally. Thus, we used the MFI structure with some Ti replacing Si as the calculation model for TS‐1, and placed a single‐layer Mn‐N‐C structure near the Ti site to simulate the TS‐1@Mn‐N‐C interface. The reaction pathway involves sequential steps of H_2_O adsorption/oxidation to H_2_O_2_ formation (Figure 5b), and subsequent *η*‐Ti‐OOH generation (Figure 5c). As revealed, the H_2_O_2_ generation is thermodynamically favorable on the Mn‐N‐C site, in comparison with the apparent energy barriers of TS‐1 (2.56 eV) and TS‐1@Mn‐N‐C sites (1.48 eV). This pronounced energetic advantage aligns with prior DFT studies highlighting the superior activity of well‐defined M‐N‐C sites for the initial 2e^−^ WOR step to H_2_O_2_ [49], and quantitatively explains why the Mn‐N‐C site is the preferred location for H_2_O activation and H_2_O_2_ generation. Following H_2_O_2_ formation, its transfer and activation at the Ti site to form *η‐*Ti‐OOH is critical [50], in which the Lewis acidity of Ti^4+^ significantly weakens the O─O bond in H_2_O_2_, lowering the activation energy for oxygen transfer compared to free H_2_O_2_ [51]. The differential charge density analysis of H_2_O_2_ adsorption (Figure S28) visually demonstrated the cooperative interactions facilitated by the intimate TS‐1 and Mn‐N‐C interface in the TS‐1@Mn‐N‐C model. Importantly, DFT calculations showed a lower energy barrier for *η*‐Ti‐OOH formation at the TS‐1@Mn‐N‐C interface (0.17 eV) compared to that on pristine TS‐1 (0.48 eV) (Figure 5c), where the adjacent Mn‐N‐C site promotes the direct generation of the *η*‐Ti‐OOH structure from H_2_O_2_ on the Ti site, minimizing energy loss through intermediate states or inefficient H_2_O_2_ diffusion. These results are strongly supported by our experimental observations, where the TS‐1@Mn‐N‐C catalyst showed a visible increase of epoxide yield (∼20%) compared to separating TS‐1 in electrolyte under identical conditions (Figure 5a). Under operational (anodic) conditions, the Mn site is predominantly occupied by oxygen or hydroxyl groups (e.g., as Mn‐OH or Mn‐O), rendering it less active toward H_2_O_2_ decomposition [52], as evidenced by in situ Raman analysis (Figure 4d). The proximity and electronic coupling at the intimate TS‐1/Mn‐N‐C interface, combined with the oxidized Mn site (Mn‐OH), likely facilitate a more direct pathway toward *η*‐Ti‐OOH. Specifically, the Mn‐OH group could act as a proton acceptor or participate in hydrogen bonding, lowering the energy barrier for H_2_O_2_ adsorption and activation directly on the adjacent Ti site. This interfacial synergy facilitates efficient H_2_O_2_ activation at adjacent Ti sites and minimizes the need for long‐range diffusion of H_2_O_2_ through the bulk electrolyte.

It is worth noting that the EXAFS analysis also reveals a weak Mn‐related second‐shell contribution, suggesting that a minor fraction of Mn species may experience secondary Mn–Mn interactions or form small Mn ensembles. Such local structural perturbations may modulate the electronic structure and intrinsic reactivity of the single‐atom Mn sites and thus potentially influence H_2_O activation and H_2_O_2_ generation. Nevertheless, the present study primarily focuses on the synergistic coupling between the Mn‐containing shell and Ti sites at the TS‐1/Mn‐N‐C interface. In the Mn‐containing comparison systems used to establish the interface effect, the Mn‐related secondary structural contribution is not the distinguishing variable; therefore, it does not change our conclusion that the enhanced epoxidation mainly originates from the spatially confined coupling of H_2_O_2_ generation and Ti‐mediated H_2_O_2_ activation. A systematic investigation of the individual roles of Mn nuclearity and coordination environment in H_2_O_2_ generation will be pursued in future studies.

### 2.3 Substrate Scope of Electrochemical Epoxidation

**FIGURE 6:**
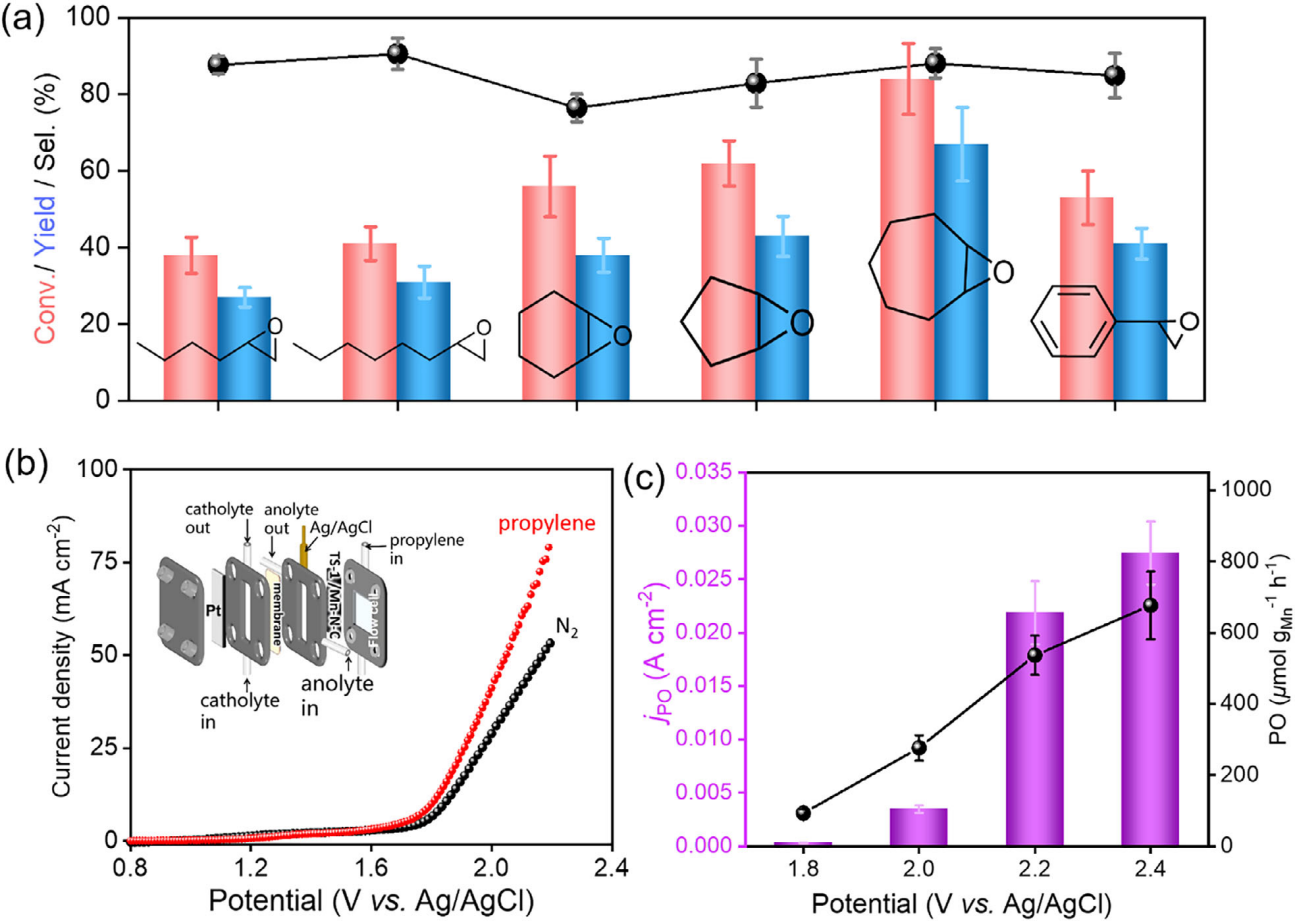
(a) Conversion of various substrates and the corresponding yield and selectivity of epoxides on TS‐1@Mn‐N‐C. (b) Scheme and polarization curve for electrochemical propylene oxidation in a flow cell configuration, and (c) Partial current density and production rate of PO at varying potentials.

In a further set of experiments, we examined the efficacy of TS‐1@Mn‐N‐C for the electrosynthesis of value‐added epoxides from various cyclic and chain olefins. It showed good compatibility for cyclic olefin substrates, including 1‐hexene, 1‐octene, cyclohexene, cyclopentene, cycloheptene, cyclooctene and styrene, with high selectivity and yield (Figure 6a and Table S2). For chain olefins (e.g., 1‐hexene and 1‐octene), TS‐1@Mn‐N‐C exhibited relatively lower activity, likely due to the absence of ring strain, a factor that typically distorts and activates the C═C bond. We further targeted propylene, a fundamental C_3_ chain olefin feedstock of paramount industrial importance, for further investigation. Its epoxidation product, propylene oxide (PO), is a multi‐million‐ton commodity chemical crucial for producing polyurethanes, propylene glycol, and other derivatives [53]. The electrochemical oxidation of propylene was carried out on TS‐1@Mn‐N‐C in a flow cell (Figure 6b). As schematically shown, the flow paths of propylene and electrolyte are on respective sides of the gas diffusion electrode, so that propylene and electrolyte are well mixed at the gas–liquid interface. Therefore, the overall reaction current is greatly improved compared with the H‐type electrolytic cell. The current with propylene was higher than that with N_2_, associated with the favorable epoxidation compared to the oxygen evolution reaction (OER). More importantly, the catalytic performance was quantitatively evaluated in terms of F.E. and yield (Figure 6c). The F.E. of PO increased steadily from 1.8 V to a maximum at 2.2 V vs. Ag/AgCl, before slightly decreasing owing to the extensive OER at 2.4 V vs. Ag/AgCl. In parallel, the PO yield showed a continuous increase with applied potential, reaching 680 µmol g_Mn_^−1^ h^−1^ at 2.4 V vs. Ag/AgCl. To further clarify whether the superior performance in the gas‐diffusion configuration originates from the core‐shell architecture rather than simply from the presence of both catalytic components, additional control electrodes with sequentially coated TS‐1 and Mn‐N‐C layers were evaluated under otherwise identical conditions (Figure S29). The sequentially coated configurations exhibited lower propylene oxidation activity than the TS‐1@Mn‐N‐C core‐shell electrode, demonstrating that intimate nanoscale integration of the two components is also critical under gas‐diffusion conditions.

## 3 Conclusion

In summary, the designed TS‐1@Mn‐N‐C core‐shell electrocatalyst successfully integrates H_2_O_2_ electrosynthesis via efficient 2e^−^ WOR on atomically dispersed Mn‐N‐C sites with subsequent in situ H_2_O_2_ activation for selective epoxidation on Ti sites into a nanostructure. TS‐1@Mn‐N‐C achieved a high yield (∼64.7%) and selectivity (∼83.0%) of cyclooctene to 1,2‐epoxycyclooctane, superior to TS‐1 and Mn‐N‐C, outperforming recent reports. Accordingly, in situ Raman and EPR spectroscopies confirmed the facilitated chemisorption and further epoxidation of cyclooctene on TS‐1@Mn‐N‐C interfaces owing to the synergistic H_2_O_2_ generation and TS‐1 epoxidation. Moreover, DFT calculations demonstrated that the TS‐1@Mn‐N‐C interface can adsorb the H_2_O_2_ molecules and facilitate the generation of *η*‐Ti‐OOH, which was conducive to the successive epoxidation. This synergistic coupling, facilitated by the intimate interfaces, enables highly efficient, selective, and stable electrochemical epoxidation of various olefins using water as the green oxygen source under mild conditions. This work provides a promising and sustainable strategy for epoxide production, aligning with green chemistry principles and offering significant advantages over conventional industrial processes. It should be noticed that the single‐atom Mn sites constitute the major local Mn environment, while the weak Mn‐related secondary coordination revealed by EXAFS may further modulate the electronic structure and intrinsic activity of the Mn sites. Elucidating the individual effects of Mn nuclearity and coordination environment on electrosynthesis will be an important direction for future investigation.

## Author Contributions

**Wenbiao Zhang**: funding acquisition, Writing – original draft, Writing – review and editing, conceptualization, software. **Guanqiao Zhang**: investigation. **Wanling Zhang**: investigation, validation. **Kun Yu**: methodology, investigation. **Di Pan**: investigation, formal analysis. **Zhuxin Gui**: investigation, data curation. **Yi Tang**: funding acquisition, writing – original draft, conceptualization, supervision, writing – review and editing. **Qingsheng Gao**: conceptualization, funding acquisition, writing – original draft, supervision, writing – review and editing.

## Funding

This work was supported by the National Natural Science Foundation of China (22501107, 22575099 and 22688201), Science and Technology Project in Guangzhou (202102070001), Fundamental and Interdisciplinary Disciplines Breakthrough Plan of the Ministry of Education of China (JYB2025XDXM802), Guangdong Province International Science and Technology Cooperation Project (2025A0505020057), Guangdong Basic and Applied Basic Research Foundation (2026A1515010789), and Science and Technology Commission of Shanghai Municipality (2024ZDSYS02).

## Conflicts of Interest

The authors declare no conflicts of interest.

## Supporting information




**Supporting File**: advs77922‐sup‐0001‐SuppMat.docx.

## Data Availability

The data that support the findings of this study are available in the supplementary material of this article.

## References

[1] A. Jalil , E. E. Happel , L. Cramer , et al., “Nickel Promotes Selective Ethylene Epoxidation on Silver,” Science 387 (2025): 869–873, 10.1126/science.adt1213. 39977491

[2] M. Chung , J. H. Maalouf , J. S. Adams , C. Jiang , Y. Román‐Leshkov , and K. Manthiram , “Direct Propylene Epoxidation Via Water Activation Over Pd‐Pt Electrocatalysts,” Science 383 (2024): 49–55, 10.1126/science.adh4355. 38175873

[3] P. Zhang , T. Wang , and J. Gong , “Advances in Electrochemical Oxidation of Olefins to Epoxides,” CCS Chemistry 5 (2023): 1028–1042, 10.31635/ccschem.023.202202643.

[4] W. R. Leow , Y. Lum , A. Ozden , et al., “Chloride‐Mediated Selective Electrosynthesis of Ethylene and Propylene Oxides at High Current Density,” Science 368 (2020): 1228–1233, 10.1126/science.aaz8459. 32527828

[5] X. Liu , Z. Chen , S. Xu , et al., “Bromide‐Mediated Photoelectrochemical Epoxidation of Alkenes Using Water as an Oxygen Source with Conversion Efficiency and Selectivity up to 100%,” Journal of the American Chemical Society 144 (2022): 19770–19777, 10.1021/jacs.2c06273. 36260532

[6] J. Herzberger , K. Niederer , H. Pohlit , et al., “Polymerization of Ethylene Oxide, Propylene Oxide, and Other Alkylene Oxides: Synthesis, Novel Polymer Architectures, and Bioconjugation,” Chemical Reviews 116 (2015): 2170–2243, 10.1021/acs.chemrev.5b00441. 26713458

[7] T. Pu , H. Tian , M. E. Ford , S. Rangarajan , and I. E. Wachs , “Overview of Selective Oxidation of Ethylene to Ethylene Oxide by Ag Catalysts,” ACS Catalysis 9 (2019): 10727–10750.

[8] S. J. Khatib and S. T. Oyama , “Direct Oxidation of Propylene to Propylene Oxide with Molecular Oxygen: A Review,” Catalysis Reviews 57 (2015): 306–344, 10.1080/01614940.2015.1041849.

[9] Q. Jing and K. D. Moeller , “From Molecules to Molecular Surfaces. Exploiting the Interplay Between Organic Synthesis and Electrochemistry,” Accounts of Chemical Research 53 (2019): 135–143, 10.1021/acs.accounts.9b00578. PMC7853972 31891254

[10] T. Wu and K. D. Moeller , “Organic Electrochemistry: Expanding the Scope of Paired Reactions,” Angewandte Chemie International Edition 60 (2021): 12883–12890, 10.1002/anie.202100193. 33768678

[11] B. A. Frontana‐Uribe , R. D. Little , J. G. Ibanez , A. Palma , and R. Vasquez‐Medrano , “Organic Electrosynthesis: A Promising Green Methodology in Organic Chemistry,” Green Chemistry 12 (2010): 2099, 10.1039/c0gc00382d.

[12] A. M. Knight , S. J. Kan , R. D. Lewis , O. F. Brandenberg , K. Chen , and F. H. Arnold , “Diverse Engineered Heme Proteins Enable Stereodivergent Cyclopropanation of Unactivated Alkenes,” ACS Central Science 4 (2018): 372–377, 10.1021/acscentsci.7b00548. PMC5879470 29632883

[13] L. L. Holbrook and H. Wise , “Electrooxidation of Olefins at a Silver Electrode,” Journal of Catalysis 38 (1975): 294–298, 10.1016/0021-9517(75)90090-1.

[14] G. R. Stafford , “The Electrogenerative Partial Oxidation of Propylene,” Electrochimica Acta 32 (1987): 1137–1143, 10.1016/0013-4686(87)80024-5.

[15] K. Otsuka , Y. Shimizu , I. Yamanaka , and T. Komatsu , “Wacker Type and ?‐Allyl Type Oxidations of Propylene Controlled by Fuel Cell System in the Gas Phase,” Catalysis Letters 3 (1989): 365–369, 10.1007/BF00771264.

[16] X.‐C. Liu , T. Wang , Z.‐M. Zhang , et al., “Reaction Mechanism and Selectivity Tuning of Propene Oxidation at the Electrochemical Interface,” Journal of the American Chemical Society 144 (2022): 20895–20902, 10.1021/jacs.2c09105. 36345048

[17] B. Gilbert , T. Cavoue , M. Aouine , et al., “Ag‐Based Electrocatalysts for Ethylene Epoxidation,” Electrochimica Acta 394 (2021): 139018, 10.1016/j.electacta.2021.139018.

[18] S. Iguchi , M. Kataoka , R. Hoshino , and I. Yamanaka , “Direct Epoxidation of Propylene with Water at a PtO x Anode Using a Solid‐Polymer‐Electrolyte Electrolysis Cell,” Catalysis Science & Technology 12 (2022): 469–473, 10.1039/D1CY01888D.

[19] K. Jin , J. H. Maalouf , N. Lazouski , N. Corbin , D. Yang , and K. Manthiram , “Epoxidation of Cyclooctene Using Water as the Oxygen Atom Source at Manganese Oxide Electrocatalysts,” Journal of the American Chemical Society 141 (2019): 6413–6418, 10.1021/jacs.9b02345. 30963761

[20] M. Chung , K. Jin , J. S. Zeng , T. N. Ton , and K. Manthiram , “Tuning Single‐Atom Dopants on Manganese Oxide for Selective Electrocatalytic Cyclooctene Epoxidation,” Journal of the American Chemical Society 144 (2022): 17416–17422, 10.1021/jacs.2c04711. PMC9523708 36098659

[21] Y. Zhao , M. Duan , C. Deng , et al., “Br−/BrO−‐Mediated Highly Efficient Photoelectrochemical Epoxidation of Alkenes on α‐Fe_2_O_3_ ,” Nature Communications 14 (2023): 1943, 10.1038/s41467-023-37620-8. PMC10082182 37029125

[22] J. Rudolph , K. L. Reddy , J. P. Chiang , and K. B. Sharpless , “Highly Efficient Epoxidation of Olefins Using Aqueous H_2_O_2_ and Catalytic Methyltrioxorhenium/Pyridine: Pyridine‐Mediated Ligand Acceleration,” Journal of the American Chemical Society 119 (1997): 6189–6190, 10.1021/ja970623l.

[23] J. Wang , G. Wu , G. Feng , et al., “Electrochemical Epoxidation of Propylene to Propylene Oxide Via Halogen‐Mediated Systems,” ACS Omega 8 (2023): 46569–46576. 10.1021/acsomega.3c05508 PMC10720275 38107883

[24] Y. Yang , J.‐L. Luo , and X.‐Z. Fu , “Water‐Oxidation Intermediates Enabling Electrochemical Propylene Epoxidation,” Chinese Journal of Structural Chemistry 43 (2024): 100269, 10.1016/j.cjsc.2024.100269.

[25] C. P. Gordon , H. Engler , A. S. Tragl , et al., “Efficient Epoxidation Over Dinuclear Sites in Titanium Silicalite‐1,” Nature 586 (2020): 708–713, 10.1038/s41586-020-2826-3. 33116285

[26] K. Dong , Y. Wang , L. Zhang , et al., “Epoxidation of Olefins Enabled by an Electro‐Organic System,” Green Chemistry 24 (2022): 8264–8269, 10.1039/D2GC02885A.

[27] X. Han , K. Wang , G. Zhang , W. Gao , and J. Chen , “Application of the Electrochemical Oxygen Reduction Reaction (ORR) in Organic Synthesis,” Advanced Synthesis & Catalysis 361 (2019): 2804–2824, 10.1002/adsc.201900003.

[28] M. C. Y. Tang , K.‐Y. Wong , and T. H. Chan , “Electrosynthesis of Hydrogen Peroxide in Room Temperature Ionic Liquids and In Situ Epoxidation of Alkenes,” Chemical Communications (2005): 1345, 10.1039/b416837b. 15742073

[29] M. Ko , Y. Kim , J. Woo , et al., “Direct Propylene Epoxidation with Oxygen Using a Photo‐Electro‐Heterogeneous Catalytic System,” Nature Catalysis 5 (2022): 37–44, 10.1038/s41929-021-00724-9.

[30] K. Yu , S. Guan , W. Zhang , et al., “Engineering Asymmetric Electronic Structure of Co─N─C Single‐Atomic Sites Toward Excellent Electrochemical H_2_O_2_ Production and Biomass Upgrading,” Angewandte Chemie International Edition 64 (2025): 202502383, 10.1002/anie.202502383. 40014009

[31] X. Hu , Z. Sun , G. Mei , X. Zhao , B. Y. Xia , and B. You , “Engineering Nonprecious Metal Oxides Electrocatalysts for Two‐Electron Water Oxidation to H_2_O_2_ ,” Advanced Energy Materials 12 (2022): 2201466, 10.1002/aenm.202201466.

[32] H. Li , M. Zhong , C. Li , Y. Ren , J. Chen , and Q. Yang , “Synthesis of CNTs@POP‐Salen Core‐Shell Nanostructures for Catalytic Epoxides Hydration,” Chemcatchem 11 (2019): 3952–3958, 10.1002/cctc.201900311.

[33] N. Jiang , Z. Zhu , W. Xue , B. Y. Xia , and B. You , “Emerging Electrocatalysts for Water Oxidation under Near‐Neutral CO_2_ Reduction Conditions,” Advanced Materials 34 (2022): 2105852, 10.1002/adma.202105852. 34658063

[34] S. T. Wu , H. L. Zhang , X. Huang , and Z. D. Wei , “Coupling Electrochemical H_2_O_2_ Production and the In Situ Selective Oxidation of Organics Over a Bifunctional TS‐1@Co–N–C Catalyst,” Chemical Communications 58 (2022): 8942–8945, 10.1039/D2CC03181G. 35861315

[35] D. Pan , L. Kong , H. Zhang , Y. Zhang , and Y. Tang , “TS‐1 Synthesis via Subcrystal Aggregation: Construction of Highly Active Hydrogen‐Bonded Titanium Species for Alkene Epoxidation,” ACS Applied Materials & Interfaces 15 (2023): 28125–28134, 10.1021/acsami.3c04487. 37260285

[36] L. Cumaranatunge and W. N. Delgass , “Enhancement of Au Capture Efficiency and Activity of Au/TS‐1 Catalysts for Propylene Epoxidation,” Journal of Catalysis 232 (2005): 38–42, 10.1016/j.jcat.2005.02.006.

[37] X. Wen , C. Yu , B. Yan , et al., “Morphological and Microstructural Engineering of Mn‐N‐C with Strengthened Mn‐N Bond for Efficient Electrochemical Oxygen Reduction Reaction,” Chemical Engineering Journal 475 (2023): 146135, 10.1016/j.cej.2023.146135.

[38] G. Chen , Y. An , S. Liu , et al., “Highly Accessible and Dense Surface Single Metal FeN_4_ Active Sites for Promoting the Oxygen Reduction Reaction,” Energy & Environmental Science 15 (2022): 2619–2628, 10.1039/D2EE00542E.

[39] P. Cui , L. Zhao , Y. Long , L. Dai , and C. Hu , “Carbon‐Based Electrocatalysts for Acidic Oxygen Reduction Reaction,” Angewandte Chemie 62 (2023): 202218269. 10.1002/anie.202218269 36645824

[40] S. Tian , X. Jia , L. Wang , et al., “The Mn‐Catalyzed Paired Electrochemical Facile Oxychlorination of Styrenes via the Oxygen Reduction Reaction,” Chemical Communications 55 (2019): 12104–12107, 10.1039/C9CC06746A. 31544175

[41] Z. Mtshali , K. G. von Eschwege , and J. Conradie , “Electrochemical Study of the Mn (II/III) Oxidation of Tris (Polypyridine) Manganese (II) Complexes,” Electrochimica Acta 391 (2021): 138965.

[42] X. Lin , Z. Zhou , Q.‐Y. Li , et al., “Direct Oxygen Transfer From H_2_O to Cyclooctene over Electron‐Rich RuO_2_ Nanocrystals for Epoxidation and Hydrogen Evolution,” Angewandte Chemie International Edition 61 (2022): 202207108, 10.1002/anie.202207108. 35789523

[43] Y. Zhang , A. Iqbal , J. Zai , et al., “Bromine and Oxygen Redox Species Mediated Highly Selective Electro‐Epoxidation of Styrene,” Organic Chemistry Frontiers 9 (2022): 436–444, 10.1039/D1QO01588E.

[44] Y. Zhao , C. Deng , D. Tang , et al., “α‐Fe_2_O_3_ as a Versatile and Efficient Oxygen Atom Transfer Catalyst in Combination with H_2_O as the Oxygen Source,” Nature Catalysis 4 (2021): 684–691, 10.1038/s41929-021-00659-1.

[45] C. Burlet and Y. Vanbrabant , “Study of the Spectro‐Chemical Signatures of Cobalt–Manganese Layered Oxides (Asbolane–Lithiophorite and Their Intermediates) by Raman Spectroscopy,” Journal of Raman Spectroscopy 46 (2015): 941–952, 10.1002/jrs.4755.

[46] X. Li , K. Wu , S. Chen , et al., “Hydrogen Peroxide‐Mediated Tandem Catalysis For Electrifying Chemical Synthesis,” Chem Catalysis 4 (2024): 100997.

[47] D. T. Bregante , A. M. Johnson , A. Y. Patel , et al., “Cooperative Effects between Hydrophilic Pores and Solvents: Catalytic Consequences of Hydrogen Bonding on Alkene Epoxidation in Zeolites,” Journal of the American Chemical Society 141 (2019): 7302–7319, 10.1021/jacs.8b12861. 30649870

[48] J. Yang , S. Liu , Y. Liu , et al., “Review and perspectives on TS‐1 catalyzed propylene epoxidation,” Iscience 27 (2024): 109064, 10.1016/j.isci.2024.109064. PMC10875142 38375219

[49] L. Xie , W. Zhou , Z. Qu , et al., “Effect of Molecular Size on the Electrocatalytic Activity of M‐N 4 ‐C catalysts for ORR, OER, and HER,” Journal of Materials Chemistry A 13 (2025): 1788–1795, 10.1039/D4TA07699K.

[50] S.‐K. Zhang , Y. Feng , A. Elgazzar , et al., “Interfacial Electrochemical‐Chemical Reaction Coupling for Efficient Olefin Oxidation to Glycols,” Joule 7 (2023): 1887–1901, 10.1016/j.joule.2023.06.022.

[51] D. T. Bregante , J. Z. Tan , R. L. Schultz , et al., “Catalytic Consequences of Oxidant, Alkene, and Pore Structures on Alkene Epoxidations within Titanium Silicates,” ACS Catalysis 10 (2020): 10169–10184, 10.1021/acscatal.0c02183.

[52] B. Battistella and K. Ray , “O_2_ and H_2_O_2_ Activations at Dinuclear Mn and Fe Active Sites,” Coordination Chemistry Reviews 408 (2020): 213176, 10.1016/j.ccr.2019.213176.

[53] C. Allard , “Alternative Propylene Oxide Production,” Nature Reviews Materials 8 (2023): 77, 10.1038/s41578-023-00532-6.

