# Supplementary material for: Interface‐Enhanced Electrochemical Epoxidation Over a Core‐Shell Nanozeolite TS‐1@Mn‐N‐C Catalyst
Source: Adv Sci (Weinh). 2026 Sep 27:e77922. Online ahead of print. doi: 10.1002/advs.77922 (PMC13616335; doi:10.1002/advs.77922)
Supplement: Supplementary file 1 [file ADVS-9999-e77922-s001.docx]

Supporting Information

Interface-Enhanced Electrochemical Epoxidation over a Core-Shell Nanozeolite TS-1@Mn-N-C Catalyst

Wenbiao Zhang,^[a,b]^ Guanqiao Zhang,^[a]^ Wanling Zhang,^[a]^ Kun Yu,^[a]^ Di Pan,^[b]^ Zhuxin Gui,^[b]^ Yi Tang*^[b]^ and Qingsheng Gao*^[a]^

Dr. W. Zhang, G. Zhang, W. Zhang, Dr. K. Yu, and Prof. & Dr. Q. Gao

College of Chemistry and Materials Science, State Key Laboratory of Bioactive Molecules and Druggability Assessment, and Guangdong Basic Research Center of Excellence for Natural Bioactive Molecules and Discovery of Innovative Drugs, Jinan University, Guangzhou 510632, China
E-mail: tqsgao@jnu.edu.cn

Dr. W. Zhang, D. Pan, Z. Gui, and Prof. & Dr. Y. Tang
Department of Chemistry, College of Smart Materials and Future Energy, State Key Laboratory of Porous Materials for Separation and Conversion, Shanghai Key Laboratory of Molecular Catalysis and Innovative Materials, and Laboratory of Advanced Materials, Fudan University, Shanghai 200433, China

E-mail: yitang@fudan.edu.cn

Experimental Section

*Preparation of TS-1:* TS-1 zeolite was hydrothermally synthesized by using tetraethyl orthosilicate (TEOS) as the silicon source, tetrabutyl orthotitanate (TBOT) as the titanium source, and tetrapropylammonium hydroxide (TPAOH) as the structure-directing agent. 5.324 g of TEOS was added to 7.934 g of 25% TPAOH solution and stirred vigorously for about 1 h until completely hydrolyzed to obtain solution A. Then, 0.217 g of TBOT was dissolved into 1 mL isopropyl alcohol to obtain solution B.

Under stirring, the solution B was slowly added to the solution A that had been cooled to room temperature. After aging at room temperature for 18 h, the obtained clear liquid was placed in an autoclave reactor, followed by hydrothermal treatment at 140 ^o^C for 48 h. Afterward, the product was recovered by centrifugation, washed with distilled water, dried at 80 ^o^C overnight, and finally calcined at 550 ^o^C for 6 h under air flow.

*Synthesis of TS-1@PDA:* 200 mg of TS-1 was added into a 100 mL conical flask with 50 mL of HCl-Tris (pH 8.5, 50 mM) solution. The mixture was stirred ultrasonically at 6 °C for 20 min, and then 200 mg of dopamine was added. The reaction solution was continuously stirred for 48 h. The obtained TS-1@PDA were collected by centrifugation at a low speed of 6000 rpm and washed three times with ethanol and deionized water, respectively, and then vacuum-dried at 60 °C overnight.

*Synthesis of TS-1@Mn-N-C catalyst:* 200 mg of prepared TS-1@PDA and 50 mg of manganese acetylacetonate were mixed in a mortar and ground for 30 min. The obtained mixture was pyrolyzed in a tube furnace under an Ar atmosphere. The temperature was first ramped to 200 °C at 5 °C min^-1^ and held for 2 h, then increased to 800 °C at 2 °C min^-1^ and maintained for another 2 h. After cooling to room temperature, the samples were taken out and ground for 20 min and washed with 0.5 M H_2_SO_4_ for 12 h in an oil bath at 80 °C. The TS-1@Mn-N-C was finally obtained by suction filtered and washing with deionized water until pH neutral, vacuum drying at 60 °C overnight. TS-1@N-C was obtained by the above method without adding manganese.

*Characterizations.* SEM images were measured with an FEI Nova NanoSEM 450 field emission scanning electron microscope. TEM and HRTEM images were recorded via a JEM-2100F microscope. The adsorption-desorption isotherms were acquired on a Quantachrome iQ-2 instrument by Ar-sorption experiments at 87 K after outgassing at 573 K for 7 h, and textural parameters were calculated by Brunauer-Emmett-Teller (BET) method and t-plot method. The pore size distributions were also calculated by the NLDFT method. Powder XRD patterns were performed on Bruker D8 Advance X-ray diffractometer with a Cu Kα radiation source (λ=1.5418 Å). ICP-AES measurements were conducted on an iCAP6300 spectrometer for Mn concentration. XPS tests were carried out on a Kratos Axis Ultra DLD spectrometer equipped with Al Kα radiation. The binding energy was calibrated concerning the C 1s level 284.8 eV of contaminated carbon. Conversions and selectivity of all substrates were determined by gas chromatography (GC-2014C SHIMADZU) with an HP-5 capillary column (30 m × 0.25 mm, 0.25 μm) and gas chromatography-mass spectrometry (GC-MS QP2010 SE SHIMADZU) with SH-Rxi-5Sil MS capillary column (30 m × 0.25 mm, 0.25 μm).

*Electrochemical measurements:* To prepare working electrodes, 10 mg of electrocatalysts and 100 μL of 5 wt% Nafion solution were dispersed in 1.0 mL water–ethanol with a V_water_/V_ethanol_ ratio of 3:1 and then the mixture was vigorously sonicated for at least 30 min to get a homogeneous ink. The as-obtained ink (300 μL) was loaded onto pre-treated carbon cloth (1.5 cm × 1.5 cm) and dried naturally in air. The working electrode was directly subjected to the epoxidation, which was conducted on a CHI 660E electrochemical workstation (Chenhua Instruments Shanghai Co, Ltd, China), within a gas-tight two-compartment electrochemical cell equipped with a piece of a proton-exchange membrane (Nafion 117) as the separator. A Ag/AgCl and a platinum electrode were used as the reference and counter electrodes, respectively. Linear sweep voltammetry (LSV) was conducted in an undivided cell with and without 10 mM cyclooctene in 40 mL 0.1 M LiClO_4_ mixture solutions (pH 6.8, V_H2O_/V_EtOH_=3:1). Water electrooxidation to H_2_O_2_ was conducted in a divided H-cell in 10 mL 0.1 M LiClO_4_ aqueous solutions. All catalytic measurements were independently repeated 3 times, and the data are presented as mean ± standard deviation.

*Product analysis:* 2 mL of the anolyte was extracted with 2 mL of CH_2_Cl_2_, and the organic phase was dried over anhydrous Na_2_SO_4_. Finally, the extracted organic phase was passed through a membrane filter and analyzed by GC and GC-MS to determine the components of products and conversion and selectivity. The conversion, F.E., selectivity and yield were calculated as follows:

$$\text{F}\text{.}\text{E}\text{.}\text{ }\left( \text{\%} \right)\text{=}\frac{\text{2}\text{n}\text{epoxideF}}{\text{Q}}\text{×100\%}$$

$$\text{Conversion (\%)=}\frac{\text{C}\text{0}\text{-}\text{C}\text{t}}{\text{C}\text{0}}\text{×100\%}$$

$$\text{Yield}\text{ }\left( \text{\%} \right)\text{=}\frac{\text{C}_{\text{epoxide}}}{\text{C}\text{0}}\text{×100\%}$$

$$\text{S}\text{electivity }\left( \text{\%} \right)\text{=}\frac{\text{C}_{\text{epoxide}}}{\text{C}_{\text{epoxide}}\text{+}\text{C}_{\text{ketone}}}\text{×100\%}$$

where *C*_0_ and *C*_t_ are the initial and final concentrations of cyclooctene, respectively. The *C*_epoxide_ was the amount of cyclooctene oxide and the *C*_ketone_ was the amount of cyclooctanone. The Q was the actual amount of electricity consumed during the reaction and F was the Faradaic constant of 96487 C mol^−1^.

The concentration of H_2_O_2_ was measured by cerium sulfate titration method according to the following equation:

2Ce^4+^+H_2_O_2_=2Ce^3+^+2H^+^+O_2_

The yellow Ce^4+^ would convert into colorless Ce^3+^, and the change of color was determined by Uv-Vis to quantify the amount of consumed Ce^4+^, so that obtaining the concentration of H_2_O_2_.

The production rate, F.E. of H_2_O_2_ were calculated as follows:

$$\text{F}\text{.}\text{E}\text{.}\text{ }\left( \text{\%} \right)\text{=}\frac{\text{2}\text{n}\text{H}\text{2}\text{O}\text{2}\text{F}}{\text{Q}}\text{×100\%}$$

$$\text{rate }\left( \text{μmol }\text{h}^{\text{-1}}\text{ }\text{cm}^{\text{-2}} \right)\left( \text{\%} \right)\text{=}\frac{\text{C}_{\text{H2O2}}\text{ × }\text{V}_{\text{electrolyte}}\text{ (μmol)}}{\text{time(h) ×electrode area}\text{ }\text{(}\text{cm}^{\text{-2}}\text{)}}$$

*DFT Calculation:* All density functional theory (DFT) calculations were carried out by using the Gaussian 16 package. Geometry optimizations were performed with PBE0 functional, def2-SVP basis set for Ti, Mn atoms and 6-31G* basis set for the other atoms. Vibrational frequency calculations at the same level were performed to verify that each stationary point reached a minimum (no imaginary frequency) or a transition state (only one imaginary frequency). Single point calculations were further calculated at PBE0/def2-TZVP level. The dispersion corrections with Grimme's D3 method were taken into consideration in all calculations. The charge density difference (CDD) was performed with the Multiwfn software and the VMD package.

**Figure S1.** (a) UV-Visible spectra of different concentration of H_2_O_2_ in 0.5 mM cerium sulfate solution. (b) The linear fitting curves between absorbance and concentration of the cerium sulfate solution. (c) Water electrooxidation to H_2_O_2_ at different M-N-C catalysts for 3 h.

**Figure S2.** FT-IR spectra of TS-1, TS-1@PDA, TS-1@N-C and TS-1@Mn-N-C.

**Figure S3.** TGA curves of TS-1, TS-1@PDA, TS-1@N-C and TS-1@Mn-N-C under a N_2_ flow.

**Figure S4.** SEM and TEM images of (a) TS-1 and (b)TS-1@Mn-N-C.

**Figure S5.** HAADF-TEM images of Mn-N-C.

**Figure S6.** XRD patterns of TS-1@N-C, TS-1@Mn-N-C and Mn-N-C.

**Figure S7.** Mn 2p XPS profiles of TS-1@N-C, TS-1@Mn-N-C and Mn-N-C.

**Figure S8.** N 1s XPS profiles of TS-1@N-C, TS-1@Mn-N-C and Mn-N-C.

**Figure S9.** O 1s XPS profiles of TS-1@N-C, TS-1@Mn-N-C and Mn-N-C.

**Figure S10.** (a) Pressure resistance and (b) pressure conductivity of TS-1, TS-1@Mn-N-C and Mn-N-C.

**Figure S11.** (a) Ar isothermal adsorption and desorption curves and (b) NLDFT pore-contribution of TS-1 and TS-1@Mn-N-C.

**Figure S12.** Mn K-edge EXAFS (points) and the fit (line) for (a) Mn foil, (b) MnO_2_, (c) MnPc, (d) Mn-N-C and (e) TS-1@Mn-N-C, shown in k3 weighted k-space.

**Figure S13.** Nyquist plots collected on (a,b) TS-1@Mn-N-C and (c,d) Mn-N-C in 0.1 M LiClO_4_ (V_H2O_:V_EtOH_ = 3:1) (a,c) with and (b,d) without 10 mM cyclooctene at different potentials.

**Figure S14.** Bode plots for EIS on (a,b) TS-1@Mn-N-C and (c,d) Mn-N-C in 0.1 M LiClO_4_ (V_H2O_:V_EtOH_ = 3:1) (a,c) without and (b,d) with 10 mM cyclooctene at different potentials.

**Figure S15.** Time-dependent composition of anolyte on TS-1@Mn-N-C at 2.0 V vs. Ag/AgCl.

**Figure S16.** Current density during the recycling test for electrochemical epoxidation.

**Figure S17.** XRD patterns of TS-1@Mn-N-C before and after the electrochemical test.

**Figure S18.** (a) HR-TEM, (b) HAADF-STEM, and (c) EDS mapping of the spent TS-1@Mn-N-C.

**Figure S19.** (a) Mn K-edge XANES, (b) FT-EXAFS and (c) the corresponding WT of TS-1@Mn-N-C before and after the electrochemical test.

**Figure S20.** Time-dependence H_2_O_2_ concentration of electrolyte on TS-1@Mn-N-C and Mn-N-C with and without cyclooctene in 0.1M LiClO_4_.

In the absence of cyclooctene, Mn-N-C continuously accumulated H_2_O_2_, reaching approximately 31 mM after 3 h, while TS-1@Mn-N-C also exhibited continuous H_2_O_2_ accumulation, reaching approximately 17 mM after 3 h. In contrast, in the presence of 10 mM cyclooctene, the H_2_O_2_ concentration in the TS-1@Mn-N-C system remained substantially lower, reaching only approximately 4 mM after 3 h. The pronounced suppression of the steady-state H_2_O_2_ concentration in the presence of cyclooctene indicates that electrogenerated H_2_O_2_ is rapidly consumed in the subsequent epoxidation reaction. Notably, this effect is much more pronounced for TS-1@Mn-N-C than for Mn-N-C, supporting the role of the adjacent TS-1 component in promoting efficient H_2_O_2_ utilization.

**Figure S21.** Electrochemical epoxidation of cyclooctene on different M-N-C at 2.0 V vs. Ag/AgCl.

**Figure S22.** (a) H_2_O_2_ production rate and (b) epoxide yield in different electrolytes.

**Figure S23.** Effect of TS-1 size on the epoxidation activity of TS-1@Mn-N-C at 2.0 V vs. Ag/AgCl.

**Figure S24.** Electrochemical epoxidation activity of different zeolite@Mn-N-C and TiO_2_@Mn-N-C composites.

**Figure S25.** Correlation of epoxidation currents with (a) H_2_O and (b) cyclooctene concentration on TS-1@Mn-N-C at 2.0 V vs. Ag/AgCl.

**Figure S26.** Comparison of thermocatalysis and electrocatalysis for cyclooctene epoxidation.

**Figure S27.** The performance of conventional TS-1-H_2_O_2_ thermo-catalysis and electrocatalysis in this work after five consecutive runs without electrolyte replacement.

**Figure S28.** Differential charge density of H_2_O_2_ adsorption on (a) TS-1/Mn-N-C (front), (b) TS-1/Mn-N-C (side), (c) TS-1 (front) and (d) TS-1(side).

**Figure S29.** Current density and production rate of PO on different coating style between TS-1 and Mn-N-C at 2.0V vs. Ag/AgCl.

To further clarify whether the superior performance in the gas-diffusion configuration originates from the core-shell architecture rather than simply from the presence of both catalytic components, additional control electrodes with sequentially coated TS-1 and Mn-N-C layers were evaluated under otherwise identical conditions. The sequentially coated configurations exhibited lower propylene oxidation activity than the TS-1@Mn-N-C core-shell electrode, demonstrating that intimate nanoscale integration of the two components is also critical under gas-diffusion conditions.

**Table S1.** EXAFS fitting parameters at the Mn K-edge (Ѕ02=0.90*)

| Sample | Path | C.N. | R (Å) | σ^2^×10^3^ (Å^2^) | ΔE (eV) | R factor |
| --- | --- | --- | --- | --- | --- | --- |
| Mn foil | Mn-Mn | 12* | 2.71±0.03 | 10.1±3.6 | 7.5±3.9 | 0.015 |
| MnO_2_ | Mn-O | 6.1±0.8 | 1.88±0.01 | 1.1±0.8 | 2.4±2.2 | 0.015 |
|  | Mn-Mn | 3.2±1.6 | 3.04±0.02 | 2.2±2.1 | -4.4±0.6 |  |
|  | Mn-Mn | 7.0±1.6 | 3.43±0.01 | 1.7±1.2 | -0.3±2.1 |  |
| MnPc | Mn-N | 4* | 1.95±0.01 | 2.0±0.8 | 2.5±1.4 | 0.018 |
| Mn-N-C | Mn-N | 4.6±1.0 | 2.17±0.02 | 13.0±0.9 | -1.2±2.2 | 0.025 |
|  | Mn-Mn | 1.46±2.1 | 2.72±0.02 | 20.0±0.9 | -7.5±0.9 |  |
| TS-1@Mn-N-C | Mn-N | 4.3±1.1 | 1.95±0.02 | 4.8±0.8 | -0.8±2.9 | 0.030 |
|  | Mn-Mn | 1.39±1.1 | 2.94±0.05 | 5.4±0.9 | -9.7±7.5 |  |
| TS-1@Mn-N-C  (spent catalyst) | Mn-N | 4.6±1.2 | 1.95±0.02 | 5.7±0.8 | -3.4±3.0 | 0.028 |
|  | Mn-Mn | 1.05±0.9 | 2.95±0.04 | 4.6±0.9 | -8.0±8.0 |  |

*C.N.*: coordination numbers; *R*: bond distance; *σ*^2^: Debye-Waller factors; Δ*E*: the inner potential correction. *R* factor: goodness of fit. * fitting with fixed parameter.

The obtained XAFS data was processed in Athena (version 0.9.26) for background, pre-edge line and post-edge line calibrations. Then Fourier transformed fitting was carried out in Artemis (version 0.9.26). The k^3^ weighting, k-range of ~3 - ~12 Å^-1^ and R range of 1 ~3 Å were used for the fitting. The four parameters, coordination number, bond length, Debye-Waller factor and E_0_ shift (CN, R, σ^2^, ΔE_0_) were fitted without anyone was fixed, constrained, or correlated.

**Table S2.** Comparison of catalyst performances for electrocatalytic epoxidation using water as the oxygen source.

| Catalysts | Type | Potential | Current density  (mA cm^-2^) | Yield  (%) | Selectivity  (%) | Stability  (h) | Structure | Reference |
| --- | --- | --- | --- | --- | --- | --- | --- | --- |
| TS-1@Mn-N-C | EC | 2.0 V vs. Ag/AgCl | ~6.5 | 64.7 | 83.0 | 15×6h |  | This work |
| MnO_x_ | EC | 1.45 V vs. Fc/Fc+ | 2.5 | 28 | 72.1 | 4 |  | ^[1]^ |
| Ir_single_-MnO_x_ | EC | 1.45 V vs. Fc/Fc+ | 10.5 | <6.5% | ~70% | N.A. |  | ^[2]^ |
| RuO_2_/N_0.12_C | EC | 1.2 V vs. Ag/AgCl | 4.5 | 36 | 90 | 18 |  | ^[3]^ |
| GF-CoS_2_/CoS | EC | 4.6-7.3 V vs SCE | 0.03 | 0 | 0 | 5 |  | ^[4]^ |
| α-Fe_2_O_3_ | PEC | 0.37 V vs. Fc/Fc+ | 0.5 | 7.15 | 8.7 | 2 |  | ^[5]^ |
| Fe_2_O_3_/Br | PEC | 0.15 V vs. Fc/Fc+ | 1.5 | 62 | 62 | 2.5 |  | ^[6]^ |

**Table S3.** Scope of substrates catalyzed by TS-1@Mn-N-C.

| Substrate | Main products yield^b^ (%) | Conv. | Sel. (%) |
| --- | --- | --- | --- |
|  | 2.1^a^, 2.7^b^, 27.1^c^  2.9^a^, 3.3^b^, 3.8^c^ | 6^a^  6^b^  38^c^ | 87 |
|  | 31^a^  2^a^ | 41^a^ | 90 |
|  | 38^c^14^c^ | 56^c^ | 76 |
|  | 43^c^ 5^c^ | 62^c^ | 83 |
|  |  67^a^ 12^a^ | 84^a^ | 88 |
|  | 41^c^9^c^ | 53^c^ | 85 |

a Reaction conditions: substrate (10 mM), LiClO_4_ (0.1 M), ethanol/H_2_O (1:3, 40 mL), TS-1@Mn-N-C anode, and Pt foil cathode with 2.0 V versus Ag/AgCl for 6 h at room temperature in an H-type cell.

b 2.0 V versus Ag/AgCl for 12 h.

c The concentration of substrate was reduced to 5 mM and the working potential was 2.0 V versus Ag/AgCl for 12h

**References**

[1]	K. Jin, J. H. Maalouf, N. Lazouski, N. Corbin, D. Yang, K. Manthiram, *J. Am. Chem. Soc.* **2019**, *141*, 6413-6418.

[2]	M. Chung, K. Jin, J. S. Zeng, T. N. Ton, K. Manthiram, *J. Am. Chem. Soc.* **2022**, *144*, 17416-17422.

[3]	X. Lin, Z. Zhou, Q. Y. Li, D. Xu, S. Y. Xia, B. L. Leng, G. Y. Zhai, S. N. Zhang, L. H. Sun, G. Zhao, *Angew. Chem. Int. Ed.* **2022**, *134*, e202207108.

[4]	Y. Zhang, A. Iqbal, J. Zai, S.-Y. Zhang, H. Guo, X. Liu, I. ul Islam, H. Fazal, X. Qian, *Org. Chem. Front.* **2022**, *9*, 436-444.

[5]	Y. Zhao, C. Deng, D. Tang, L. Ding, Y. Zhang, H. Sheng, H. Ji, W. Song, W. Ma, C. Chen, *Nat. Catal.* **2021**, *4*, 684-691.

[6]	Y. Zhao, M. Duan, C. Deng, J. Yang, S. Yang, Y. Zhang, H. Sheng, Y. Li, C. Chen, J. Zhao, *Nat. Commun.* **2023**, *14*, 1943.
